# Long-term survival and clinical outcomes of delayed chest closure following lung transplantation

**DOI:** 10.1007/s00595-024-02821-1

**Published:** 2024-03-28

**Authors:** Takashi Hirama, Miki Akiba, Masahiro Ui, Saori Shibata, Fumiko Tomiyama, Tatsuaki Watanabe, Yui Watanabe, Hirotsugu Notsuda, Takaya Suzuki, Hisashi Oishi, Hiromichi Niikawa, Masafumi Noda, Yoshinori Okada

**Affiliations:** 1 Department of Thoracic Surgery, Institute of Development, Aging and Cancer, Tohoku University, 4-1 Seiryomachi, Sendai, Miyagi 980-8575 Japan; 2 Division of Organ Transplantation, Tohoku University Hospital, 1-1 Seiryomachi, Sendai, Miyagi 980-8574 Japan

**Keywords:** Lung transplant, Delayed chest closure, Survival, Outcome, Chronic lung allograft dysfunction, Physical performance

## Abstract

**Purposes:**

Delayed chest closure (DCC) is a widely accepted procedure in the context of lung transplantation (LTx); yet there are few reports detailing its long-term survival and clinical outcomes.

**Methods:**

We reviewed the medical records of recipients who underwent deceased-donor lung transplantation (LTx) at Tohoku University Hospital. Long-term survival, including overall survival, freedom from chronic lung allograft dysfunction (CLAD), and CLAD-free survival and the clinical outcomes of graft function and physical performance and constitution were reviewed in recipients with DCC.

**Results:**

Between 2009 and 2022, 116 patients underwent LTx, 33 of whom (28.4%) required DCC. The intra—and post-operative courses of the recipients who required DCC were more complicated than those of the recipients who underwent primary chest closure (PCC), with frequent volume reduction surgery and longer periods of invasive mechanical ventilation. Pulmonary vascular disease was considered a risk factor for these complications and DCC. Nonetheless, long-term survival and graft functions were comparable between the DCC and PCC groups. The physical performance and constitution of recipients who required DCC continued to improve, and by 2 years after transplantation, exhibited almost no difference from those who underwent PCC.

**Conclusions:**

In view of the profoundly complicated intra- and post-operative courses, DCC should be performed cautiously and only when clinically indicated, despite which it can result in equivalent long-term survival and acceptable outcomes to PCC.

**Supplementary Information:**

The online version contains supplementary material available at 10.1007/s00595-024-02821-1.

## Introduction

With recent advances in operative techniques and significant improvement of post-transplant management, lung transplantation (LTx) has become a promising treatment for various pulmonary diseases at their end stage. In low-volume transplant centers or countries with a significant lack of organ donations, efforts have been made to promote transplant opportunities by innovating surgical skills and increasing access to transplantation through the use of oversized grafts or marginal donor lungs with pulmonary edema. Delayed chest closure (DCC) is a specialized technique employed in the transplantation of oversized grafts, grafts with pulmonary edema, and for hemodynamic instability following lung transplantation. DCC allows fluid to be removed from edematous lungs and reduces the risk of primary graft dysfunction by preventing injured lungs or a dilated right ventricle from being subjected to excessive compression [1–3]. Despite DCC being a widely accepted procedure in the context of LTx, there have been few reports detailing long-term survival and clinical outcomes. In this study, we reviewed the multiple survivals of LTx recipients who underwent DCC followed by clinical outcomes as favorable as those who underwent primary chest closure (PCC) after LTx.

## Methods

Recipients who underwent deceased-donor LTx at Tohoku University Hospital (TUH) between January 1, 2009, and April 30, 2022, were included in the retrospective cohort, with follow-up ending in July 30, 2022. The study protocol was approved by the institutional review boards at both TUH (2021-1-476) and the Japan Organ Transplant Network (2022-35-13). Living-donor LTx recipients were excluded from the study. The donor selection and allocation system in Japan have been described previously [4, 5]. Immunosuppression, histocompatibility testing, anti-microbial prophylaxis and overall management after transplantation have also been described [6–10].

The primary objective of this study was to compare the long-term survival, freedom from chronic lung allograft dysfunction (CLAD), and CLAD-free survival of LTx recipients who underwent DCC, with those of recipients who underwent PCC. CLAD was diagnosed when an irreversible drop of ≥ 20% in the forced expiratory volume in the first second (FEV1) from the baseline was confirmed twice at least 3 months post-transplant [11]. The secondary objective was to review the trends of FEV1 as a measure of graft function, physical performance with respect to a 6 min walking distance, and physical constitution in terms of body-mass index (BMI). These variables were assessed at 3 and 6 months, and then annually, after transplantation.

During the study period, three attending surgeons performed a mean of eight transplantations annually in the period between 2009 and 2015 and four surgeons performed a mean of eight transplantations annually between 2016 and 2022. Japanese transplant centers perform single rather than bilateral LTx to maximize LTx opportunities through donor sharing [5]. At our center, bilateral LTx is performed through a clamshell incision, whereas single LTx is performed through a unilateral anterolateral incision and transverse sternotomy or posterolateral thoracotomy based on the patient’s condition and the transplant side.

The decision to perform DCC is made by the transplant surgeons responsible for the operation, taking into account factors such as hemodynamic stability if there is excessive bleeding during extracorporeal circulation (ECLS), if pulmonary edema is observed when using marginal lungs or if there is prolonged ischemia, and/or graft size-mismatch associated with height disparities in donor-recipient pairs or thoracic deformities in recipients. In patients with pulmonary vascular disease, particularly in the presence of advanced pulmonary hypertension, there is a potential risk of left cardiac failure developing, leading to primary graft dysfunction. This risk arises from the swift reduction of pulmonary vascular resistance and an increase in left ventricular filling pressure and cardiac output. Consequently, a postoperative strategy has been implemented at our transplant center, involving the use of DCC. This strategy aims to prevent excessive compression on injured lungs or a dilated right ventricle and promotes the normalization of cardiac hemodynamics, thereby reducing the incidence of primary graft dysfunction. Notably, our transplant center maintains a lower threshold for implementing DCC in patients with pulmonary vascular disease. In conflict resolution, we often prioritize DCC approaches over trying to address PCC issues.

**Fig. 1:**
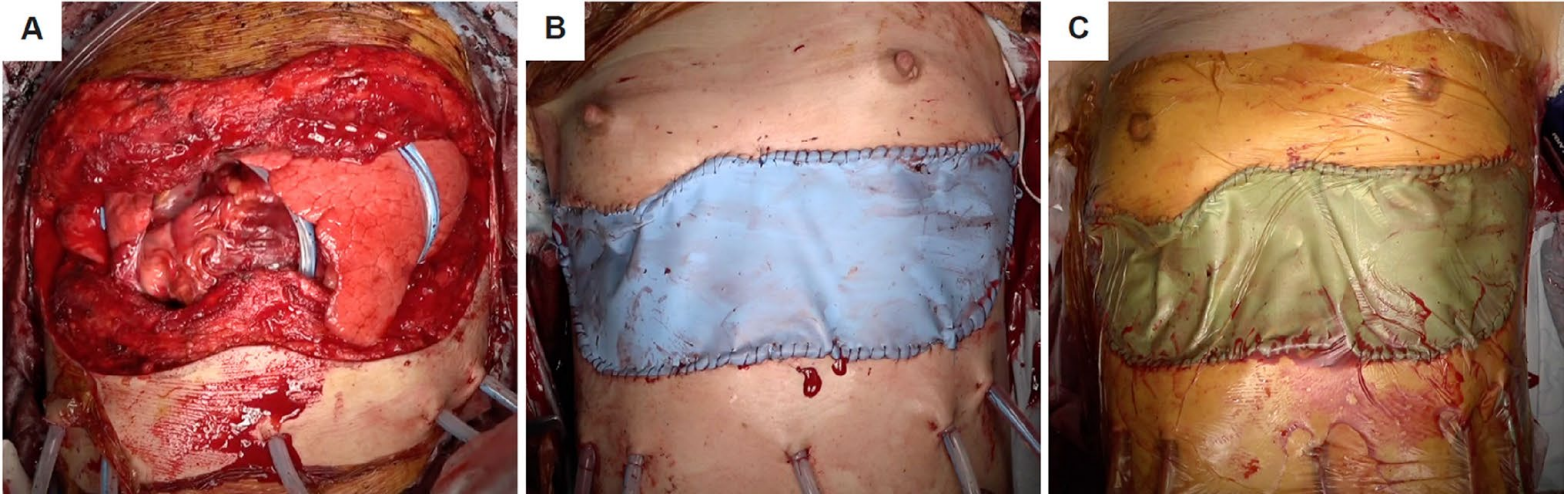
Photographic images of the delayed chest closure procedure **A** the skin incision before chest closure, **B** attachment of the Esmarch bandage and **C** an iodine-impregnated drape covering the chest.

A trimmed sterile Esmarch bandage (Hogy Medical Co., Ltd., Tokyo, Japan) was attached to the edge of a skin incision with a 2–0 polypropylene (Prolene, Ethicon, Somerville, NJ) running suture and covered with an iodine-impregnated drape (3 M^™^ Ioban^™^ Special Incise Drape, 3 M Japan, Tokyo, Japan) **(**Fig. 1**)**. On returning to the intensive care unit (ICU), recipients were diuresed aggressively and coagulopathy was controlled. During open-chest management, our center uses Vancomycin with a target trough level of between 15 and 20 µg/mL or Daptomycin 4 mg/kg and Micafungin 150 mg until the sternum is closed.

The decision to close the chest following DCC varies based on the recipient's clinical condition. The timing of chest closure surgery is decided after confirming stable hemodynamics, the absence of indications for reopening to treat hemorrhage, the absence of severe primary graft dysfunction, and sufficient responsiveness to diuretics for weight control. Chest closure surgeries are typically scheduled within the available weekday time slots.

The use of intraoperative ECLS, including off-pump, extracorporeal membrane oxygenation (ECMO), and cardiopulmonary bypass (CPB), were reviewed as independent variables [12]. Surgical site infection (SSI) was defined as [1] a positive culture from fluid drained from the thoracotomy incision associated with cutaneous fluctuance, erythema, or induration, or [2] a positive culture from pleural fluid in the first 3 months after transplantation [3]. Donors’ lungs were considered extended if any one of the following criteria was met: age greater than 55 years, smoking history greater than 20 pack-years, the presence of chest radiographic infiltrate, PaO2 of less than 300 mmHg on 100% oxygen with 5 cm H2O positive end-expiratory pressure, or purulent secretions on bronchoscopy [13].

The variables between DCC and PCC are expressed as medians (interquartile range [IQR]) or as the actual count (percentage) as appropriate, and the difference was analyzed by the Mann–Whitney U test for continuous variables and the chi-square or Fisher’s exact tests for categoric variables. The risk factors associated with DCC were estimated by a logistic regression model. Variables plausibly associated with DCC and outcome were selected [14]. The KaplanMeier method was used to model time-to-event outcomes, and differences across groups were calculated by the log-rank test. *P*-values of < 0.05 were considered significant. Changes in follow-up variables after transplantation were analyzed by mixed effects models. Statistical analyses and graph generation were performed with GraphPad Prism 10 (GraphPad Software, Inc., La Jolla, CA) and mixed effects models with R version 4.3.1.

## Results

Between 2009 and 2022, 116 patients underwent LTx at TUH, and 33 (28.4%) of these patients required DCC while the remaining majority underwent PCC (Table 1**)**. The LTx recipients who underwent DCC were younger (median age, 40 years (IQR 30–49) vs. 48 years (IQR 40–54), *p* = 0.004 and had a lower body weight (median BMI, 16.8 (IQR 15.0–20.6) vs. 19.5 (IQR 15.0–23.5), *p* = 0.008), than those who underwent PCC. The most common indication for LTx was pulmonary vascular disease (45.5%) in the DCC patients, whereas it was obstructive lung disease (47.0%) in the PCC patients (*p* < 0.0001). Bilateral LTx was the predominant procedure for the DCC patients (90.9%, *p* < 0.0001), whereas single LTx was generally performed for the PCC patients (78.3%). DCC took place in a relatively proportional manner: 19 in 59 (32.3%) from 2009 to 2015 and 14 in 57 (24.7%) from 2016 to 2022. The proportion of extended donors at our transplantation center was 62.9%, with 78.8% in the DCC group and 56.6% in the PCC group, and a higher rate of extended donors in the DCC group (p = 0.033). Details for each criterion for extended donors are outlined in Supplemental data 1. The multivariate analysis identified the following risk factors for DCC: pulmonary vascular disease (odds ratio (OR) 4.281, 95% CI 1.330–14.36 compared with other LTx indications) and ischemic time (OR 1.01, 95% CI 1.001–1.009), as presented in Table 2.

**Table 1:** Characteristics of the patients who underwent delayed chest closure (*n* = 33) versus those who underwent primary chest closure (*n* = 83)

	Total	Delayed chest closure	Primary chest closure	*p-value*
	*n* = 116	*n* = 33	*n* = 83	
Recipient age, median (IQR)	46 (35–52)	40 (30–49)	48 (40–54)	0.004
Recipient sex, male (%)	51 (44.0%)	15 (45.5%)	36 (43.4%)	0.839
BMI (kg/m^2^), median (IQR)	18.2 (16.2–22.1)	16.8 (15.0–20.6)	19.5 (15.0–23.5)	0.008
Recipient predicted VC (mL), median (IQR)	3369 (2973–4256)	3373 (2941–4335)	3365 (2991–4200)	0.562
Supplemental oxygen, *n* (%)	108 (93.1%)	33 (100%)	75 (90.4%)	0.103
Waiting time (day), median (IQR)	886 (621–1277)	959 (681–1401)	824 (589–1165)	0.196
LTx indication, *n* (%)				<0 .0001
Pulmonary vascular disease	22 (19.0%)	15 (45.5%)	7 (8.4%)	
Restrictive lung disease	34 (29.3%)	7 (21.2%)	27 (32.5%)	
Obstructive lung disease	41 (35.3%)	2 (6.1%)	39 (47.0%)	
Suppurative lung disease	12 (10.3%)	7 (21.2%)	5 (6.0%)	
Allogeneic lung disease	7 (6.0%)	2 (6.1%)	5 (6.0%)	
LTx procedure, *n* (%)				< 0.0001
Single	68 (58.6%)	3 (9.1%)	65 (78.3%)	
Bilateral	48 (41.4%)	30 (90.9%)	18 (21.7%)	
Transplant year, *n* (%)				0.414
Between 2009 and 2015	59 (43.7%)	19 (57.6%)	40 (39.2%)	
Between 2016 and 2022	57 (42.2%)	14 (42.4%)	43 (42.2%)	
Donor age, median (IQR)	44 (33–53)	44 (31–53)	44 (34–53)	0.931
Donor sex, male (%)	58 (50.0%)	19 (57.6%)	39 (47.0%)	0.303
Donor predicted VC (mL), median (IQR)	3494 (2973–4504)	3993 (2981–4564)	3492 (2970–4470)	0.562
Extended donors, *n* (%)	73 (62.9%)	26 (78.8%)	47 (56.6%)	0.033

*BMI* body-mass index, *IQR* interquartile range, *LTx* lung transplant, *VC*: vital capacity

**Table 2:** Risk factors associated with delayed chest closure in the logistic regression model

	Univariate			Multivariate		
	Odds ratio	95% CI	*p-value*	Odds ratio	95% CI	*p-value*
Recipient age	0.96	0.93–0.99	0.009			
Recipient sex: male vs. female	0.92	0.41–2.09	0.839			
LTx indication: pulmonary vascular disease vs others	9.05	3.33–26.89	< 0.0001	4.28	1.330–14.36	0.016
LTX procedure: bilateral vs. single	36.11	11.28–163.2	< 0.0001			
Ischemic time	1.01	1.00–1.01	< 0.0001	1.01	1.001–1.009	0.010
Intraoperative blood loss	1.00	1.00–1.00	0.010			

*LTx* lung transplant, *CI* confidence interval

Although an equivalent fraction of oversized graft was used in both the DCC and PCC groups (63.6% vs. 60.2%, *p* = 0.735), the difference in donor-recipient height was significant in the DCC group, with a median gap of 4.65 cm (IQR 0.82–9.79) vs. 0.23 cm (IQR − 0.41–7.42) in the PCC group (*p* = 0.007) (Supplemental data 2 and Table 3). The intra- and post-operative courses were complicated in the LTx recipients who underwent DCC, with nearly half requiring volume reduction surgery (53.1%) and most requiring tracheostomy (84.4%), versus 6.0% and 28.9%, respectively, for the PCC patients (both at *p* < 0.0001). In volume reduction surgery, the resection locations (with duplications) were as follows: right upper lobe (45.5%), right middle lobe (72.7%), right lower lobe (9.1%), upper segments of the left upper lobe (50.0%), lingular segments of the left upper lobe (72.7%), and left lower lobe (18.2%). Categorized by procedure, 68.2% underwent wedge resection, 18.2% underwent lobectomy, and 13.6% underwent combined wedge resection and lobectomy. As anticipated, the operation time, ischemic time, duration of invasive mechanical ventilation until the first attempt at extubation, and ICU stay were substantially longer for the DCC patients (all *p* < 0.0001). Intraoperative ECLS use, requirement for CPB support and the CPB time for LTx recipients with DCC was more significant than for those with PCC (all *p* < 0.0001). SSI was more prevalent in the recipients who underwent DCC than in those who underwent PCC (*p* = 0.002), and the most common agent cultured from the SSI was the *Candida* species (data not shown). The 3-month mortality rate for the entire cohort was 6.9%. Although there was a numerical distinction between the DCC group (12.1%) and the PCC group (4.8%), no significant difference was identified.

**Table 3:** Intra- and post-operative courses of patients who underwent delayed chest closure (*n* = 33) versus those who underwent primary chest closure (*n* = 83)

	Total	Delayed chest closure	Primary chest closure	*p-value*
	*n* = 116	*n* = 33	*n* = 83	
Sex mismatch, *n* (%)	19 (16.4%)	4 (12.1%)	15 (18.1%)	0.435
Oversized graft, *n* (%)	71 (61.2%)	21 (63.6%)	50 (60.2%)	0.735
Difference in donor-recipient height (cm)	1.58 (− 3.12–8.73)	4.65 (0.82–9.78)	0.28 (-0.41–7.42)	0.007
Intraoperative ECLS use, *n* (%)	85 (73.3%)	31 (93.9%)	54 (65.1%)	0.002
Intraoperative CPB use, *n* (%)	31 (26.7%)	20 (60.6%)	11 (13.3%)	< .0001
Intraoperative CPB time (min), median (IQR)	491 (417–650)	594 (473–797)	442 (374–524)	0.0005
Volume reduction, n (%)	22 (19.0%)	17 (53.1%)	5 (6.0%)	< .0001
Operation time (min), median (IQR)	499 (394–823)	805 (651–1100)	440 (375–616)	< .0001
Ischemic time (min), median (IQR)	516 (447–683)	681 (545–752)	490 (434–573)	< .0001
Blood loss (mL), median (IQR)	1217 (541–4696)	4689 (1221–10,501)	875 (435–2797)	< .0001
Duration of delayed chest closure (days), median (IQR)		3.5 (3–4)		
Surgical site infection, *n* (%)	8 (6.9%)	6 (18.2%)	2 (2.4%)	0.002
Invasive mechanical ventilation (day), median (IQR)	10 (3–29)	31 (19–41)	4 (2–17)	< .0001
ICU stay (day), median (IQR)	16 (8–36)	39 (21–52)	11 (6–29)	< .0001
Tracheostomy, *n* (%)	52 (44.8%)	28 (84.8%)	24 (28.9%)	< .0001
Continuous renal replacement therapy, *n* (%)	25 (21.6%)	11 (33.3%)	14 (16.9%)	0.052
Three-month mortality, *n* (%)	8 (6.9%)	4 (12.1%)	4 (4.8%)	0.221

Sex mismatch: male to female or female to male; Oversized graft: ratio of donor/recipient in FVC ≥ 1; ECLS use: the intraoperative use of either ECMO or CPB

*CPB *cardiopulmonary bypass, *ICU* intensive-care unit, *IQR* interquartile range; *ECLS* extracorporeal life support, *ECMO* extracorporeal membrane oxygenation, *LTx* lung transplant, *VC* vital capacity

**Fig. 2:**
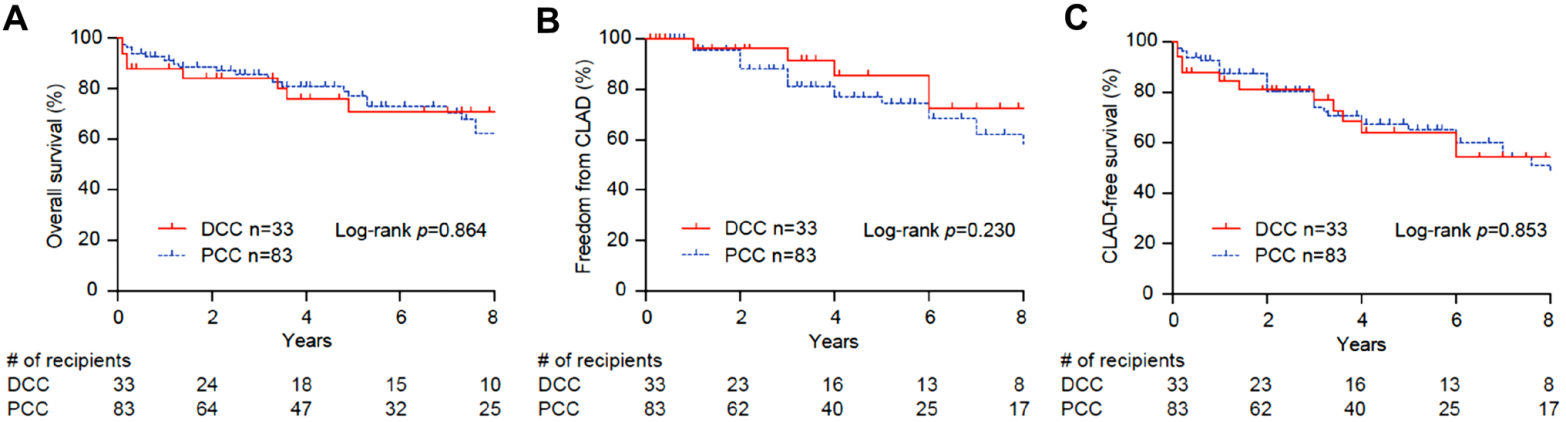
Kaplan–Meier analysis of the lung transplant recipients who underwent delayed chest closure (DCC) (*n* = 33) versus those who underwent primary chest closure (PCC) (*n* = 83) **A** Overall survival (event = death), **B** freedom from chronic lung allograft dysfunction (CLAD) (event = diagnosis of CLAD), and (C) CLAD-free survival (event = death or diagnosis of CLAD) every 2 years after transplantation. The number of patients at risk is indicated according to time.

**Fig. 3:**
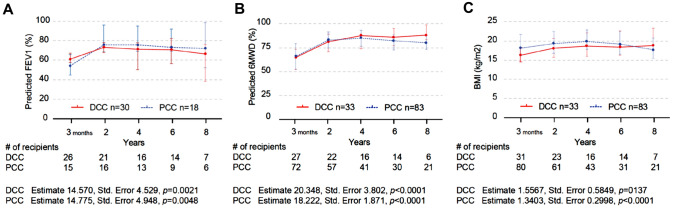
Trends of variables after transplantation in recipients who underwent (DCC) (*n* = 33) versus those who underwent primary chest closure (PCC) (*n* = 83) The trend in (A) forced expiratory volume in the first second (FEV1), B percentage predicted 6 min walk distance (6MWD), and (C) body-mass index (BMI) (kg/m^2^) at 3 months and then every 2 years after transplantation. FEV1 was analyzed in recipients who underwent bilateral LTx (30 vs. 18). Changes in follow-up data between 3 months and 2 years after transplantation were analyzed by Linear mixed effects models. The parameter estimate, standard error (Std. Error), and *p*-value are displayed below the number of patients.

The clinical research on LTx has revealed several approaches to analyze the probability of survival: overall survival, freedom from CLAD, and CLAD-free survival. Although the intra- and post-operative timeframes involved a profoundly difficult transition in DCC, survival did not differ between the two groups (Fig. 2). The overall survival 2 years and 8 years after transplantation for the DCC and PCC groups was comparable (84.4% vs. 88.6%, and 70.8% vs. 62.4% respectively). Furthermore, freedom from CLAD 2 years and 8 years after transplantation, no matter whether the recipients had undergone DCC or PCC, was similar (96.2% vs. 88.0% and 72.5% vs. 58.4%, respectively) and the same applied to CLAD-free survival (81.0% and 80.3% vs. 54.2% and 48.2%, respectively). Finally, graft function, as indicated by FEV1, improved significantly in both the DCC and PCC groups from 3 months to beyond 2 years after transplantation (Fig. 3). The physical performance of recipients, using the 6 min walk distance as the metric, whether they had undergone DCC or PCC, showed an upward trend, with no significant difference between the two groups. Likewise, an increase in body weight, based on BMI, was noted in both the DCC and PCC groups during the period spanning from 3 months to beyond 2 years after transplantation, with recipients in both groups achieving a similar physique.

## Discussion

Although DCC following LTx is becoming increasingly common in clinical practice, there are few studies on the long-term survival, graft function, and physical recovery after such a complex surgical procedure. To our knowledge, five studies comparing the outcomes of DCC with those of PCC have been published. Force et al. presented a seminal work documenting the risk of DCC [15]. Shigemura et al. analyzed that risk in a large cohort and presented a technical approach to prevent possible issues after DCC [2]. Aguilar et al. focused on SSI after DCC [3], and Rafiroiu et al. reported on the long-term survival and graft function of recipients who had undergone DCC [16]. Yeginsu et al. reported on the clinical outcomes of DCC in Turkey, together with a literature review [17]. As noted in the previous reports, the intra- and post-operative timeframes were arduous when DCC was related to an increased risk of surgical site infection [3], 30 day mortality [2], and primary graft dysfunction [16]. In addition to the above risks, our analysis compared the numerous negative impacts of DCC on post-transplant management (including more frequent volume reduction surgery and tracheostomy and longer use of invasive mechanical ventilation and ICU stay), with the outcomes of PCC. Nonetheless, the long-term survival and graft functions of the DCC and PCC groups at our center were comparable. Moreover, the physical performance and constitution of recipients who underwent DCC improved consistently and, 2 years after LTx, exhibited almost no difference from those of the recipients who underwent PCC. Thus, while DCC should be performed cautiously and only when clinically indicated, considering these features and the complicated postoperative course, it can have acceptable outcomes similar to those associated with PCC.

The favorable survival of our DCC group despite such complicated comorbidity raises the inference that early clinical management is likely to play an essential role in outcomes. Although the vacuum-assisted closure technique or negative-pressure wound therapy has become extensively utilized for SSI, antimicrobial therapy should be considered crucial in the early management of DCC. At our center, 18.2% (6 / 33) of DCC patients and 2.4% (2 / 83) of PCC patients experienced SSI (*p* = 0.002) requiring Vancomycin and Micafungin, and the long-term survival rates and functional changes were comparable between the groups. Similarly, Rafiroiu et al. reported SSI in 15.2% (7/ 46) of their DCC patients vs. 13.0% (6 /46) of their PCC patients (*p* = 0.764), which was managed with Piperacillin/Tazobactam, Vancomycin, and Micafungin post-operatively [16], and the 5 year survival did not differ significantly between the two groups. Yeginsu et al. reported that SSI developed in 25% (4 /16) of their DCC patients vs. 7.1% (2 in 28) of their PCC patients (*p* = 0.169), when broad-spectrum antibacterial and antifungal treatments had been administered [17], and the median survival (13 months) for the DCC patients did not differ from that for the PCC patients (13 months vs. 16 months, respectively) (*p* = 0.300). Although the employment of antimicrobial regimens may vary across transplant centers, intervention with antimicrobial therapy in the early management of DCC is believed to have the potential for improving outcomes.

At our center, nearly 25% of patients underwent DCC following LTx, which was a higher proportion than in previous reports [2, 15, 16]. Because of the severe donor shortage in Japan, with only 0.61 donations per million, Japanese transplant candidates face considerable issues: an extremely prolonged waiting time of over 900 days and a waitlist mortality as high as 50% [5]. To address the issue, Japanese transplant centers carry out single, rather than bilateral, LTx and attempt to use even marginal donor lungs for patients to increase the transplant opportunity [18]. In this context, DCC could be an alternative strategy to maximize the use of donated lungs when PCC cannot be administered safely because of oversized lungs or a marginal graft with pulmonary edema or intraoperative injuries. Our data support that the long-term survival and clinical outcomes are equivalent in patients who have undergone DCC and those who have undergone PCC, which is promising for low-volume transplant centers or countries with severe donor shortages wishing to expand transplant opportunities.

As described in methods, our transplant center tends to implement DCC more readily in patients with pulmonary vascular disease. Consequently, the DCC group may exhibit a higher prevalence of pulmonary vascular disease, leading to an increased frequency of bilateral LTx and potentially a greater proportion of younger patients. To address this concern, we conducted propensity score (PS) matching with LTx indication, LTx procedure, and age as covariates to standardize recipient backgrounds that might influence the outcomes of the DCC and PCC groups (Supplemental data 3). Supplemental Table 3-1 demonstrates that through matching, the differences in patient characteristics between the DCC and PCC groups were eliminated. In the analysis of this patient cohort presented in Supplemental Table 3-2, significant differences in most intra- and post-operative course variables disappeared, suggesting a strong influence of LTx indication, LTx procedure, and/or age. Conversely, variables such as the length of invasive mechanical ventilation, ICU stay, and tracheostomy are likely outcomes attributed to DCC rather than to patient backgrounds. Regarding the long-term outcomes illustrated in Supplemental Fig. 3-1, no significant differences were observed in either group, indicating that the LTx indication, LTx procedure, age, and even DCC may not exert an influence on long-term outcomes. However, considering the limited number of cases in the 19:19 analysis, drawing definitive conclusions is implausible. From the perspective of intra- and post-transplant management including DCC, it is evident that pulmonary vascular disease constitutes a distinct disease category. To improve the perioperative outcomes of LTx, additional analyses in larger studies will be necessary.

One of the limitations of this study is the selection bias related to the study sample, which, in being a retrospective sample from a single center, does not fully represent the Japanese population. The lower threshold for implementing DCC in pulmonary vascular disease at our center is associated with a potential bias in the DCC group, characterized by a higher prevalence of pulmonary vascular disease, an increased frequency of bilateral lung transplants, and possibly a higher proportion of younger patients. To overcome this, a multicenter study over all of Japan would be of great interest to understand the long-term survival and clinical outcomes of LTx recipients who have undergone DCC. Moreover, survival and outcomes were not able to be validated by multivariate analysis because of the limited sample size and the Kaplan–Meier method, given its univariate nature, is insufficient to evaluate the factors that contribute to prolonging survival and improving physical performance. Moreover, an adequate sample size is necessary for statistical significance and to achieve narrower CI around the estimated effect, thereby increasing the certainty of the outcome. The wide CI in the logistic regression models indicates a lack of precision in the estimated risk for DCC (Table 2). Finally, while this study presents evidence supporting favorable long-term survival and clinical outcomes of LTx recipients who underwent DCC, it does not address the analysis of healthcare resources, including the workload and time of healthcare providers, and the economic implications of prolonged ventilation, ICU stay, and dialysis requirements. These matters will be addressed in subsequent investigations.

In conclusion, the long-term survival and clinical outcomes of LTx recipients who underwent DCC were reported from a country having a severe donor shortage. In view of the profoundly complicated post-operative course, DCC should be performed cautiously and only when clinically indicated; however, it could also lead to long-term survival and outcomes as acceptable as those for patients who undergo PCC.

## Supplementary Information

Below is the link to the electronic supplementary material.

Supplementary file1 (DOCX 33 KB)

Supplementary file2 (DOCX 302 KB)

Supplementary file3 (DOCX 226 KB)

## Data Availability

The data that support the findings of this study are available on request from the corresponding author. The data are not publicly available due to privacy or ethical restrictions.
