# Supplementary material for: Long-term survival and clinical outcomes of delayed chest closure following lung transplantation
Source: Surg Today. 2024 Mar 28;54(10):1138–45. doi: 10.1007/s00595-024-02821-1 (PMC11413204; doi:10.1007/s00595-024-02821-1)
Supplement: Supplementary file 1 — Supplementary file1 (DOCX 33 KB) [file 595_2024_2821_MOESM1_ESM.docx]

**Supplemental data 1**

**Supplemental Table 1-1.** Criteria used to define extended donors

|  | **Total** | **Delayed chest closure** | **Primary chest closure** |  |
| --- | --- | --- | --- | --- |
| Extended donors | **n=116** | **n=33** | **n=83** | ***p-value*** |
| - Age >55 years | 21 (18.1%) | 6 (18.2%) | 15 (18.1%) | 0.989 |
| - Smoking >20 pack-years | 29 (25.0%) | 12 (36.4%) | 17 (20.5%) | 0.0966 |
| - Chest radiograph infiltrate | 38 (32.8%) | 15 (45.5%) | 23 (27.7%) | 0.081 |
| - PaO2/FiO2 <300 | 4 (3.4%) | 1 (3.0%) | 3 (36%) | >0.999 |
| - Purulent bronchoscopy | 19 (16.4%) | 7 (21.2%) | 12 (14.5%) | 0.4098 |

Abbreviations: PaO2/FiO2: PaO2 of less than 300 mm Hg on 100% oxygen with 5 cm H2O positive end-expiratory pressure
