# Supplementary material for: Long-term survival and clinical outcomes of delayed chest closure following lung transplantation
Source: Surg Today. 2024 Mar 28;54(10):1138–45. doi: 10.1007/s00595-024-02821-1 (PMC11413204; doi:10.1007/s00595-024-02821-1)
Supplement: Supplementary file 2 — Supplementary file2 (DOCX 302 KB) [file 595_2024_2821_MOESM2_ESM.docx]

**Supplemental data set 2**

**Supplemental Table 2-1.** Ratio of donor/recipient predicted forced vital capacity

|  | **Total** | **Delayed chest closure** | **Primary chest closure** |  |
| --- | --- | --- | --- | --- |
|  | **n=116** | **n=33** | **n=83** | ***p-value*** |
| Ratio ≥120% | 19 (16.4%) | 6 (18.2%) | 13 (15.7%) | 0.3265 |
| 120% > Ratio ≥ 110% | 23 (19.8%) | 7 (21.2%) | 16 (19.3%) |  |
| 110% > Ratio ≥ 100% | 29 (25.0%) | 8 (24.2%) | 21 (25.3%) |  |
| 100% > Ratio ≥ 90% | 30 (25.9%) | 11 (33.3%) | 19 (22.9%) |  |
| Ratio < 90% | 15 (12.9%) | 1 (3.0%) | 14 (16.9%) |  |

Abbreviation: FVC, forced vital capacity

**Supplemental Figure**


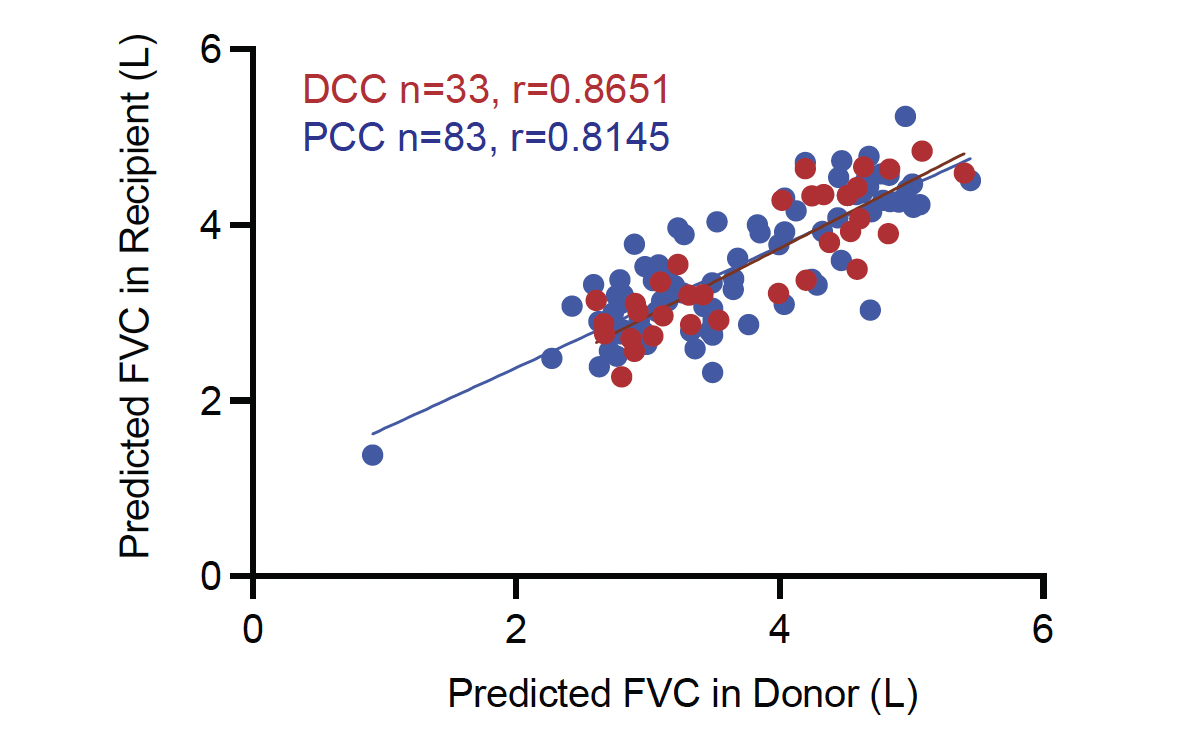


**Supplemental Figure 2-1** The correlation of predicted Forced Vital Capacity (FVC) in donors versus recipients who underwent delayed chest closure (DCC) (n = 33, depicted in red) and primary chest closure (PCC) (n = 83, depicted in blue).

Spearman’s coefficient (r) was computed to assess bivariate correlations of predicted FVC between recipients and donors
