# Supplementary material for: Long-term survival and clinical outcomes of delayed chest closure following lung transplantation
Source: Surg Today. 2024 Mar 28;54(10):1138–45. doi: 10.1007/s00595-024-02821-1 (PMC11413204; doi:10.1007/s00595-024-02821-1)
Supplement: Supplementary file 3 — Supplementary file3 (DOCX 226 KB) [file 595_2024_2821_MOESM3_ESM.docx]

**Supplemental data 3**

Method

A lower threshold for implementing Delayed Chest Closure (DCC) in pulmonary vascular disease at our center created potential bias in the DCC group, characterized by a higher prevalence of pulmonary vascular disease, an increased frequency of bilateral lung transplants, and possibly a higher proportion of younger patients. To mitigate this, we conducted propensity score (PS) matching through 1:1 non-replacement extraction, employing nearest-neighbor matching with LTx indication (pulmonary vascular disease), LTx procedure (bilateral transplant), and Age as covariates. The objective was to standardize recipient backgrounds that might influence intra- and post-operative courses, as well as long-term outcomes in both groups. Subsequently, we compared the intra- and post-operative courses, as well as long-term outcomes in 19 matched pairs between the DCC and PCC groups. The propensity score estimation involved logistic regression analysis, with the assignment variable being the DCC or PCC group.

**Supplemental Table 3-1** Characteristics of patients who underwent delayed chest closure (n = 19) versus those who underwent primary chest closure (n  = 19): After propensity score matching

|  | **Total** | **Delayed chest closure** | **Primary chest closure** |  |
| --- | --- | --- | --- | --- |
|  | **n=38** | **n=19** | **n=19** | ***p-value*** |
| Recipient age, median (IQR) | 41 (26.8-48) | 40 (30-47) | 45 (24-50.5) | 0.872 |
| Recipient sex, male (%) | 17 (44.7%) | 9 (47.4%) | 8 (42.1%) | 1.000 |
| BMI (kg/m2), median (IQR) | 17.0 (15.4-20.5) | 16.8 (15.0-19.5) | 17.0 (15.5-21.3) | 0.465 |
| Recipient predicted VC (mL), median (IQR) | 3439 (3050-4336) | 3496 (3078-4335) | 3382 (3069-4323) | 0.931 |
| Supplemental oxygen, n (%) | 37 (97.4%) | 19 (100%) | 18 (94.7%) | 1.000 |
| Waiting time (day), median (IQR) | 832 (601-1131) | 918 (632-1067) | 769 (328-1869) | 0.603 |
| LTx indication, n (%) | |  |  | 0.886 |
| - Pulmonary Vascular Disease | 15 (39.5%) | 8 (42.1%) | 7 (36.8%) |  |
| - Restrictive Lung Disease | 3 (7.9%) | 1 (5.3%) | 2 (10.5%) |  |
| - Obstructive Lung Disease | 3 (7.9%) | 2 (10.5%) | 1 (5.3%) |  |
| - Suppurative Lung Disease | 11 (28.9%) | 6 (31.6%) | 5 (26.3%) |  |
| - Allogeneic Lung Disease | 6 (15.8%) | 2 (10.5%) | 4 (21.1%) |  |
| LTx procedure, n (%) | |  |  | 0.737 |
| - Single | 8 (21.1%) | 3 (15.8%) | 5 (26.3%) |  |
| - Bilateral | 30 (78.9%) | 16 (84.2%) | 14 (73.7%) |  |
| Transplant year, n (%) | |  |  | 0.737 |
| - between 2009-2015 | 24 (63.2%) | 13 (68.4%) | 11 (57.9%) |  |
| - between 2016-2022 | 14 (36.8%) | 6 (31.6%) | 8 (42.1%) |  |
| Donor age, median (IQR) | 42.5 (34-51.5) | 42 (33.5-51) | 43 (36-52.5) | 0.988 |
| Donor sex, male (%) | 23 (60.5%) | 11 (57.9%) | 12 (63.2%) | 1.000 |
| Donor predicted VC (mL), median (IQR) | 4120 (3009-4679) | 4201 (3162-4599) | 4038 (2887-4693) | 0.930 |
| Extended donors, n (%) | 27 (71.1%) | 16 (84.2%) | 11 (57.9%) | 0.151 |

Abbreviations: BMI: body-mass index; IQR: interquartile range; LTx: lung transplant; VC: vital capacity

**Supplemental Table 3-2.** Intra- and post-operative courses of the patients who underwent delayed chest closure (n =19) versus those who underwent primary chest closure (n  = 19): After propensity score matching

|  | **Total** | **Delayed chest closure** | **Primary chest closure** |  |
| --- | --- | --- | --- | --- |
|  | **n=38** | **n=19** | **n=19** | ***p-value*** |
| Sex mismatch, n (%) | 6 (15.8%) | 2 (10.5%) | 4 (21.1%) | 0.6599 |
| Oversized graft, n (%) | 23 (60.5%) | 12 (63.2%) | 11 (57.9%) | 0.9999 |
| Difference of donor-recipient in height (cm) | 5.243 (-1.182-9.584) | 6.434 (1.948-11.02) | 4.287 (-4.718-8.943) | 0.1814 |
| Intraoperative ECLS use, n (%) | 34 (89.5) | 19 (100%) | 15 (78.9%) | 0.1050 |
| Intraoperative CPB use, n (%) | 19 (50.0%) | 10 (52.6%) | 9 (47.4%) | 0.9999 |
| Intraoperative CPB time (min), median (IQR) | 526 (450-691) | 573 (480-746) | 491 (424-678) | 0.2767 |
| Volume reduction, n (%) | 12 (31.6) | 10 (52.7%) | 2 (10.5%) | 0.0128 |
| Operation time (min), median (IQR) | 820 (634-957) | 805 (668-884) | 827 (518-967) | 0.7786 |
| Ischemic time (min), median (IQR) | 691 (578-744) | 685 (580-738) | 712 (573-752) | 0.9602 |
| Blood loss (mL), median (IQR) | 3676 (853-9647) | 4715 (1198-10197) | 2933 (627-9506) | 0.2707 |
| Duration of delayed chest closure (days), median (IQR) |  | 4 (3-4) |  |  |
| Surgical site infection, n (%) | 4 (10.5%) | 3 (15.8%) | 1 (5.3%) | 0.6039 |
| Invasive mechanical ventilation (day), median (IQR) | 24 (6-36) | 33 (23-40) | 9 (4-30) | 0.0132 |
| ICU stay (day), median (IQR) | 32 (14-45) | 39 (25-53) | 16 (11-36) | 0.0157 |
| Tracheostomy, n (%) | 27 (71.1%) | 18 (94.7%) | 9 (47.4%) | 0.0030 |
| Continuous renal replacement therapy, n (%) | 16 (42.1%) | 8 (42.1%) | 8 (42.1%) | 0.9999 |
| Three-month mortality, n (%) | 3 (7.9%) | 1 (5.3%) | 2 (10.5%) | 0.9999 |

Sex mismatch: male to female or female to male; Oversized graft: ratio of donor/recipient in FVC ≥1; ECLS use: the intraoperative use of either ECMO or CPB.

Abbreviations: CPB: cardiopulmonary bypass, ICU: intensive-care unit; IQR: interquartile range; ECLS: extracorporeal life support; ECMO: extracorporeal membrane oxygenation; LTx: lung transplant, VC: vital capacity

**Supplemental Figure**


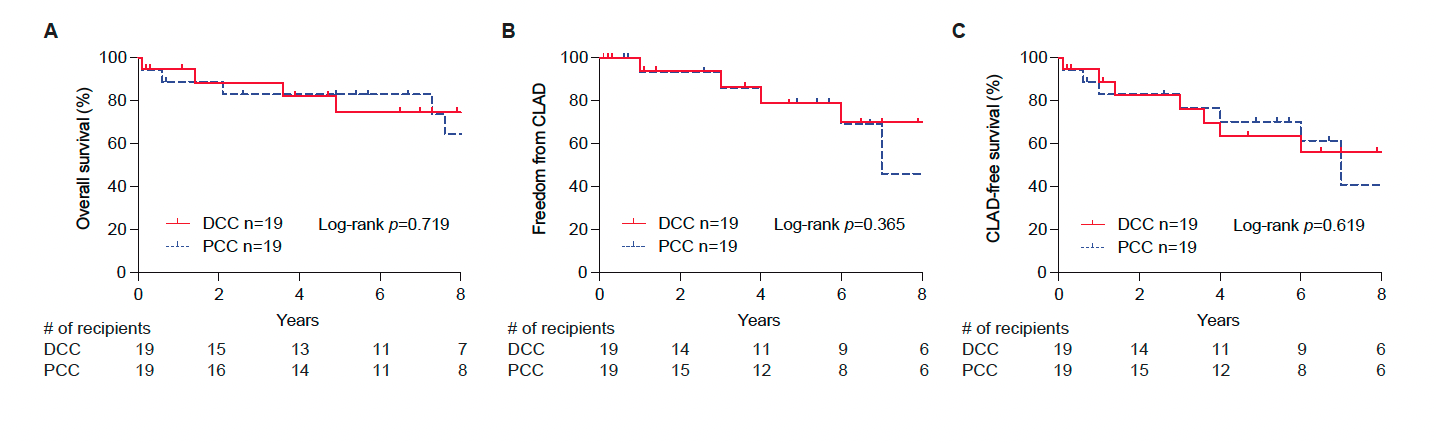


**Supplemental Figure 3-1** Kaplan-Meier analysis of lung transplant recipients who underwent delayed chest closure (DCC) (n  =  19) versus those who underwent primary chest closure (PCC) (n  = 19) : After propensity score matching

(A) Overall survival (event  = death), (B) freedom from chronic lung allograft dysfunction (CLAD) (event = diagnosis of CLAD), and (C) CLAD-free survival (event = death or diagnosis of CLAD) every 2 years after transplantation. The number of patients at risk is indicated according to time.
